# Enzymatic Functionalization of Wood as an Antifouling Strategy against the Marine Bacterium *Cobetia marina*

**DOI:** 10.3390/polym13213795

**Published:** 2021-11-02

**Authors:** Daniel Filgueira, Cristian Bolaño, Susana Gouveia, Diego Moldes

**Affiliations:** 1 CINTECX, Department of Chemical Engineering, Campus Universitario as Lagoas-Marcosende, Universidade de Vigo, 36310 Vigo, Spain; daniel.martinez@tecnalia.com (D.F.); cbolano@uvigo.es (C.B.); gouveia@uvigo.es (S.G.); 2 TECNALIA, Basque Research and Technology Alliance (BRTA), Area Anardi 5, 20730 Azpeitia, Spain; 3 Research Group of Bioengineering and Sustainable Processes, Department of Chemical Engineering, Edificio Fundición, Lagoas Marcosende s/n, University of Vigo, 36310 Vigo, Spain

**Keywords:** laccase, lauryl gallate, wood, *Cobetia marina*, antifouling

## Abstract

The protection of wood in marine environments is a major challenge due to the high sensitivity of wood to both water and marine microorganisms. Besides, the environmental regulations are pushing the industry to develop novel effective and environmentally friendly treatments to protect wood in marine environments. The present study focused on the development of a new green methodology based on the laccase-assisted grafting of lauryl gallate (LG) onto wood to improve its marine antifouling properties. Initially, the enzymatic treatment conditions (laccase dose, time of reaction, LG concentration) and the effect of the wood specie (beech, pine, and eucalyptus) were assessed by water contact angle (WCA) measurements. The surface properties of the enzymatically modified wood veneers were assessed by X-ray photoelectron spectroscopy (XPS), Fourier transform-infrared spectroscopy (FTIR). Antifouling properties of the functionalized wood veneers against marine bacterium *Cobetia marina* were studied by scanning electron microscopy (SEM) and protein measurements. XPS and FTIR analysis suggested the stable grafting of LG onto the surface of wood veneers after laccase-assisted treatment. WCA measurements showed that the hydrophobicity of the wood veneers significantly increased after the enzymatic treatment. Protein measurements and SEM pictures showed that enzymatically-hydrophobized wood veneers modified the pattern of bacterial attachment and remarkably reduced the bacterium colonization. Thus, the results observed in the present study confirmed the potential efficiency of laccase-assisted treatments to improve the marine antifouling properties of wood.

## 1. Introduction

Wood is a renewable, cheap, and biodegradable bioresource with interesting mechanical properties which make it a competitive material in construction applications. In marine environments, wood is used as a raw material for the construction of waterfront structures, e.g., groynes, jetties, dolphins [1] and classic boats. Besides, wood is used in marine platforms for the aquaculture of mollusks and crustaceous species. However, wood is highly hydrophilic and very sensitive to the attack of biological organisms [2]. These characteristics are a major challenge in marine environments, due to the regular wet conditions and the great number of living organisms in seawater. Under these conditions, all the immersed surfaces are rapidly colonized by different microorganisms leading to the formation of a biofilm, but also larger organisms could colonize the substrata after the biofilm formation. This phenomenon is commonly known as marine biofouling [3].

The biofilm formation starts with the adsorption of free organic material onto the surface of the substrata [3]. Then, bacteria and other microorganisms (e.g., diatoms, protozoa) are the first living organisms in colonizing the substrata [4]. Bacteria have the capacity to secrete extracellular polymeric substances (EPS) which are mainly composed of carbohydrates and proteins [5], forming a 3D structure of bacterial aggregates which facilitates their adhesion onto the substrata [6]. Such biofilm may change the physicochemical properties of the substrata, influencing the future adhesion of larger fouling organisms (e.g., barnacles, mollusks) [7]. A wide variety of paints and coatings have been used to limit the marine biofouling. Most of them involved the use of heavy metals (e.g., copper, zinc) or organic compounds that are toxic for fouling organisms, but also for non-target organisms such as algae, mollusks, crustaceans and fishes and they could be introduced in the food chain [8]. One of the most extended antifouling chemicals used in paints (tributyltin) was banned years ago and other hazardous treatments should be avoided to protect the marine ecosystems. Environmental factors and physicochemical properties of surfaces are the two main parameters which may favor or hinder the biofilm formation [9]. Since environmental factors are not controllable, the modification of the physicochemical properties of the substrata may disturb the pattern of bacterial colonization, minimizing the subsequent adhesion of marine fouling organisms. Based on such a mechanism, different non-biocide antifouling treatments have been proposed in the last decade [7,10]; most of them aim to interfere with the adhesion of marine microorganisms adjusting the surface free energy of the substrata between a specific range [11], which is usually controlled with hydrophobic compounds e.g., fluorinated polymers or silicones [12].

Another important issue regarding the protection of wood in marine environments is the leaching of the antifouling coatings and paints, since this release results in water pollution but also in the requirement of a new antifouling treatment. Covalent grafting of antifouling compounds onto surfaces could significantly reduce or avoid their leaching, providing stable protection against fouling organisms. Regarding wood as a material, a sustainable alternative to conventional chemical and thermal treatments is the use of laccase, which enable the stable grafting of several chemical compounds. Such lignolytic enzyme can oxidize phenolic and amine groups of a target compound [13,14], leading to the formation of radicals that may link to the aromatic structure of woody lignin by radical polymerization. By this mechanism, laccase could be used for functionalization of lignocellulosic materials or their components, mainly lignin. It is worth noting that laccase-assisted reactions are performed in mild conditions, minimizing the energy requirements. Thus, the physicochemical properties of wood can be tailored by means of an environmentally-friendly pathway. Enzymatic hydrophobization of wood veneers was successfully performed with different chemical compounds such as alkylamines [15] and fluorophenols [16]. Alkyl gallates are laccase-specific substrates with an aliphatic chain at the *para*-position that could provide stable hydrophobic properties if grafted onto wood. In fact, these compounds have been enzymatically grafted on different lignocellulosic substrates for such purpose [17,18], but also for providing antibacterial properties [19] since the aliphatic tail also presents this characteristic.

Therefore, the reaction conditions namely lauryl gallate (LG) concentration, laccase dose and time of reaction for the enzymatic hydrophobization of wood veneers were assessed in the present study. Water contact angle (WCA) measurements were carried out for the assessment of the wettability of the enzymatically-hydrophobized wood veneers. In addition, the surface of the hydrophobized wood samples was characterized by X-ray photoelectron spectroscopy (XPS) and Fourier transform infrared spectroscopy (FTIR). Finally, the potential antifouling properties of the enzymatically-hydrophobized wood veneers were studied against the marine bacterium *Cobetia marina* by means of protein measurements and scanning electron microscopy (SEM), in order to assess the capability of hydrophobic functionalization of wood as a new antifouling strategy for such material.

## 2. Materials and Methods

### 2.1. Materials

Beech (*Fagus sylvatica*), eucalyptus (*Eucalyptus globulus*) and pine (*Pinus pinaster*) wood veneers were supplied by Foresa (Caldas de Reis, Spain). Beech and eucalyptus veneers were vaporized before supplying. These wood species were selected because of their importance regarding production and commercial interest in Galicia (NW of Spain).

Commercial laccase from *Myceliophthora thermophila* (NS51003) was provided by Novozymes (Bagsværd, Denmark). The activity of the enzyme was calculated by the 2,2′-azino-bis(3-ethylbenzothiazoline-6-sulphonic acid) (ABTS) oxidation assay. One unit of activity is defined as the amount of enzyme that oxidizes 1 μmol of ABTS per minute at 25 °C and pH 7 in a 0.1 M phosphate buffer.

*Cobetia marina* (CECT4278) bacterium was purchased from the Colección Española de Cultivos Tipo—CECT (Valencia, Spain).

Bradford reactive was provided by Bio-Rad (Hercules, CA, USA).

All the other chemical reagents were acquired from Sigma-Aldrich (St. Louis, MO, USA) at reagent grade and used without any kind of purification.

### 2.2. Enzymatic Hydrophobization of Wood Veneers

Wood veneers from beech, pine or eucalyptus were cut in square plugs (50 × 50 mm), washed with distilled water at 50 °C for 30 min and oven-dried at 50 °C for 4 h. Each wood sample was then immersed in 58 mL of a phosphate buffer (pH 7, 0.1 M): acetone solution (60:40, *v*/*v*) at 50 °C. An experimental design was performed for assessing the treatment conditions which could provide the highest hydrophobic properties to wood veneers. Three levels of each of the selected parameters were chosen: laccase dose (3.36, 5.00 and 6.72 U/cm^2^ of wood), LG concentration (2.5, 5 and 10 mM) and treatment time (1, 2 and 4 h). All the possible combinations of such parameters were assayed (Table S1). The enzymatically treated samples were then dried at a room temperature (18–20 °C) for 12 h and washed with an aqueous solution of acetone (50:50, *v*/*v*) at 50 °C for 1 h. Finally, the samples were washed with distilled water three times and oven-dried at 50 °C for 4 h. Control treatments without adding laccase and/or LG were performed in parallel. All the experiments were performed in triplicate.

### 2.3. Water Contact Angle Measurements

The hydrophobization of wood veneers was assessed by water contact angle (WCA) measurements. A goniometer MobileDrop GH11 (Krüss GmbH, Hamburg, Germany) was used to measure the contact angle of one drop of distilled water on the surface of wood veneers. DSA2 software (Krüss GmbH) was utilized to analyze the drop shape. WCA measurements were performed after water drop deposition with intervals of 30 s for 5 min. A minimum of two points per each side of the wood veneers were measured.

### 2.4. X-ray Photoelectron Spectroscopy (XPS)

The surface of wood veneers was analyzed by X-ray photoelectron spectroscopy (XPS). The analyses were performed in a Thermo Scientific K-Alpha device using a monochromized Al-K-α-X-ray source (1486.6 eV). As the samples were not conductive, an electron flood gun was used to minimize surface changing. A constant analyzed energy mode (CAE) with 100 eV pass energy for survey spectra was used to perform the measurements. Neutralization of the surface charge was implemented with a low-energy flood gun (electrons in the range 0–14 eV) and a low-energy Ar ions gun. Binding energy was adjusted using C1 (BE 284.6 eV). The elemental composition of the surface of wood samples was defined plotting on the standard Scofield photoemission cross-sections. The O/C ratio was calculated by means of the survey spectra collected (from 0 to 1350 eV) which showed the components C1s and O1s.

### 2.5. Fourier Transform Infrared Spectroscopy (FTIR)

The surface of wood pieces was also studied with a 4100 Fourier transform infrared spectrometer (Jasco), equipped with an attenuated total reflectance (ATR) device. A total of 32 scans were picked between 600 and 4000 cm^−1^ with a resolution of 4 cm^−1^. The spectra were scale normalized and analyzed with OMNIC Suite software v 7.3 (Thermo Scientific, Waltham, MA, USA).

### 2.6. Stock Bacteria and Culture Conditions

Marine broth (5 g of bacteriological peptone, 3 g of yeast extract, 750 mL filtered marine water, 250 mL distilled water and pH adjusted to 7.4) was used as culture media in all assays.

*Cobetia marina* CECT4278 was provided lyophilized. It was resuspended in marine broth, which was then used for the inoculation of two flasks with 50 mL of fresh marine broth. Such culture media was incubated in an orbital shaker at 100 rpm and 27 °C for 24 h. Optical density (OD) was measured with Unicam Helios Beta spectrophotometer (Thermo Fisher Scientific) at 600 nm. When the value of OD was 1.0, 1.5 mL of bacterial culture medium was used to inoculate fresh culture media (3% of inoculum, *v*/*v*), in duplicate. After 24 h of incubation in an orbital shaker at 100 rpm and 27 °C, the culture medium was centrifuged (10,000 rpm) and stored as stock culture in aliquots of 3 mL with glycerol (30%) at −20 °C.

Working cultures were prepared in the same way, using stock cultures as inoculum but discarding the supernatant, suspending the pellet with 1.5 mL of culture media; then 750 μL of this suspension was used for inoculating 50 mL of culture media (3% of inoculum, *v*/*v*).

### 2.7. Biofilm Assay

Pine wood samples were hydrophobized according to the results obtained from the study of the treatment conditions. After the enzymatic treatment, the veneers were cut in square plugs (25 × 25 mm), dried at 50 °C for 24 h. Finally, the pine veneers were autoclaved at 121 °C for 20 min.

For the biofilm assay, each hydrophobized pine veneer was submerged in working cultures (50 mL of marine broth inoculated with *C. marina* 3% (*v*/*v*)). The flask was incubated in an agitator with orbital shaker at 100 rpm and 27 °C for 5 days. The colonized pine veneers were gently washed with distilled water to remove unattached bacteria from their surface. Control tests using untreated pine veneers and, also control test without *C. marina* were performed. Six replicas of biofilm colonization were carried out for each experiment.

### 2.8. Bacterial Surface Hydrophobicity

Surface hydrophobicity of *C. marina* was measured by means of the microbial adhesion to hydrocarbons (MATH) method. The test was performed according to Warne Zoueki et al. [20], who adapted the method previously proposed by Rosenberg et al. [21]. Firstly, 50 mL of marine broth were inoculated (3% (*v*/*v*) *C. marina*) and incubated for 24 h. Such culture was then centrifuged (8000 rpm) at 4 °C for 20 min into a Falcon tube. The supernatant was discarded, and the recovered biomass was suspended with KCl solution (150 mM, pH 7). Centrifugation and supernatant removal steps were performed twice. Then, the biomass was suspended with KCl solution (150 mM, pH 7) and diluted with the same solution in order to obtain an absorbance of 1.0 at 600 nm (ABS_i_). After that, in our experiment, 5 mL of the diluted bacterial solution were mixed with 300 μL of dodecane in an acid washed Pyrex glass tube. Subsequently, the mixture was stirred for 10 min at 27 °C and left for 15 min to separate the aqueous phase from the organic phase. The aqueous phase was recovered, and its absorbance was measured at 600 nm (ABS_f_). The partition coefficient (P) was calculated as follows:

P = 1 − (ABS_f_/ABS_i_) (1)

### 2.9. Protein Measurement

Each colonized pine veneer was immersed in 15 mL of distilled water in a 50 mL centrifuge tube. The tube content was then sonicated with a HD 2070 Sonoplus sonicator (Bandelin GmbH, Berlin, Germany) for 10 min (power 65%, cycle 70%) divided in two equal intervals of 5 min, with a pause of 2 min. After sonication, the wood samples were removed, and the aqueous solution was centrifuged at 7500 rpm for 40 min. Finally, the protein amount of the supernatant was measured using the Bradford’s method, with slight modifications: 800 μL of the supernatant were mixed with 200 μL of Bradford’s concentrated reagent and stirred with a vortex device; the mixture was left for 20 min and its absorbance was measured at 595 nm. A standard curve (5–25 mg protein/L) for protein quantification was performed using bovine serum albumin. Protein measurements of bacteria biomass on the wood veneers were performed after one, three and five days of incubation of *C. marina*.

### 2.10. SEM Analysis

Colonized pine veneers were introduced into plastic tubes with 2% of glutaraldehyde in cacodylate buffer (0.1 M, pH 7.4) and left at 4 °C for 2 h. Part of the solution was discarded avoiding the contact of the samples with atmospheric air and sodium phosphate buffer was added and the samples left for 30 min. Such a step was performed twice. Dehydration was performed with graded ethanol series, graded ethanol:amyl acetate substitution series and CO_2_ critical point drying (73 atm, 31.3 °C). Then, the samples were coated with a layer of gold (thickness: 10–20 nm) (K550X Sputter Coater, Emitech, Ashford, UK). A Philips XL30 (SEMTech Solutions, Billerica, MA, USA) scanning electron microscope (SEM) was used to perform the SEM analysis. The applied acceleration voltage was 12kW and the magnifications were 100×, 200×, 500×, 1000×, 1500× and 2500×.

## 3. Results and Discussion

### 3.1. Enzymatic Hydrophobization

Nowadays, acetylation is the only processes available at industrial scale for the manufacturing of hydrophobic wood. However, acetylation is a high-cost process with a relatively high environmental impact. Hence, the wood industry needs to develop new sustainable routes for the manufacturing of hydrophobic wood. In this sense, laccase-assisted hydrophobization is a promising pathway to achieve an efficient hydrophobization of wood with a low environmental impact but also a competitive production cost.

Several parameters of laccase-mediated hydrophobization of beech veneers, namely laccase dose, LG concentration ([LG]) and time of reaction, were assessed to optimize the enzymatic hydrophobization process on beech veneers. The hydrophobicity of the treated beech veneers is showed as a function of the water contact angle (WCA).

**Figure 1:**
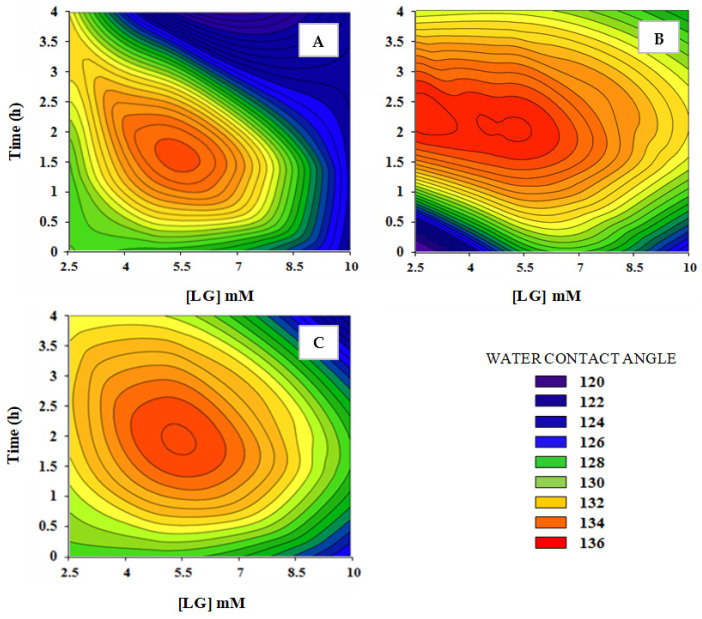
Water contact angle values after one second of drop deposition of the hydrophobized beech veneers as a function of time of reaction and lauryl gallate concentration. Laccase dose: 3.36 U/cm^2^ wood (**A**); 5.00 U/cm^2^ wood (**B**); 6.72 U/cm^2^ wood (**C**).

It was expected that long reaction time combined with high [LG] improved the hydrophobicity of the beech veneers. Nevertheless, long reaction times (higher than 3 h) did not improve the surface hydrophobicity of the wood veneers. In fact, the time of reaction necessary to achieve the highest WCA on the surface of beech veneers was between 2–3 h for the different [LG] and laccase doses studied (Figure 1). It is worth to mention that besides the grafting of LG onto the surface of beech veneers, self-polymerization of LG monomers could be expected leading to the formation of oligomers [22]. In fact, several authors have already reported the laccase-catalyzed polymerization of phenolic compounds such as epigallocatechin [23], lignin model compounds [24], or condensed tannins [25]. Hence, stable LG adsorption without correct orientation and/or grafting/adsorption of LG oligomers without proper orientation after the grafting of LG monomers could explain the lower hydrophobicity obtained after 3 h of treatment. These results allow us to set 2 h as optimum time of treatment to achieve the highest enzymatic hydrophobization of beech veneers. The [LG] that yielded the highest WCA was in the range of 5–6 mM. Moreover, [LG] higher than 6 mM reduced the hydrophobicity of the wood samples. It is likely that a [LG] higher than 5 mM favored the grafting of LG oligomers which apparently did not provide high a hydrophobization of the wood veneers. Therefore, the optimum [LG] was set at 5 mM.

Regarding the laccase dose, the results observed suggest that the higher the laccase dose the higher the WCA of the enzymatically treated beech veneers. The oxidation of the phenolic moieties of lignin present in the beech veneers enables the stable grafting of LG. Hence, the higher the laccase dose the higher the lignin oxidation and, therefore, a higher grafting of LG could be achieved. A medium laccase dose (5 U/cm^2^ of wood) was set as the optimum value considering the small differences in the WCA in the tested range. Thus, the experimental design permitted to identify the conditions to improve the hydrophobicity of treated beech veneers while minimizing the requirements of energy and both chemical and enzymatic reagents.

**Figure 2:**
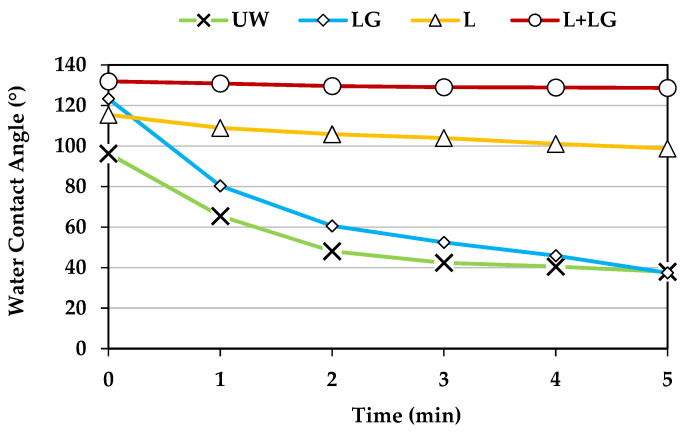
Water contact angle of beech wood veneers. Untreated (UW); treated with lauryl gallate (LG); laccase (L); and laccase and lauryl gallate (L + LG).

The WCA of the beech veneers treated with laccase and LG was compared with those samples treated only with laccase or LG. As noted in Figure 2, veneers treated with both laccase and LG showed a stable WCA and always higher than 125°, after 5 min of water drop deposition. In addition, samples treated only with laccase showed a remarkable hydrophobicity, but much lower than those samples treated with both laccase and LG. Probably, laccase oxidized the hydroxyl groups of lignin’s phenolic structures [26], leading to the formation of oxidized functionalities which, apparently, increased the hydrophobicity of lignin. Samples treated with LG alone showed a WCA similar to the observed on the untreated beech veneers. Thus, the activity of laccase was necessary to achieve a stable grafting of LG onto wood veneers surface.

**Figure 3:**
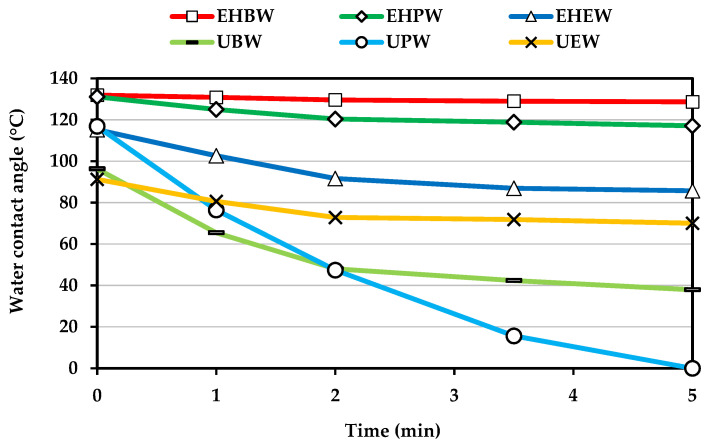
Water contact angle of wood veneers. Enzymatically hydrophobized beech wood (EHBW); pine wood (EHPW); and eucalyptus wood (EHEW). Untreated beech wood (UBW); pine wood (UPW); and eucalyptus wood (UEW).

Regarding the different wood species tested (beech, pine, and eucalyptus), enzymatically treated beech veneers showed a WCA slightly higher than pine and a much higher hydrophobicity than eucalyptus veneers (Figure 3). Nonetheless, the highest change in hydrophobicity was achieved in the enzymatically hydrophobized pine veneers, since their WCA, after 5 min of drop deposition, was about 120° whereas untreated pine veneers had already absorbed the water droplet. Enzymatically hydrophobized beech veneers showed a WCA around 70% higher than untreated samples and such gap was much lower (20%) for eucalyptus wood veneers. It is worth mentioning that LG was expected to be enzymatically grafted on lignin moieties and both eucalyptus and beech are hardwood species with a similar lignin content (≈25%). Lignin biopolymer is composed of phenylpropane units (C_6_–C_3_) which differ one each other in their methoxy substitutions on the aromatic ring, e.g., guaiacyl (G), syringyl (S) and *p*-hydroxyphenyl (H). The S/G ratio in eucalyptus wood is proximately to 6.25 which is much higher than the S/G ratio in beech wood, 0.71 [27,28]. In addition, the lignin of pine has a clear predomination of G units [29,30]. Thus, these results obtained with the WCA measurements suggest that the laccase-assisted grafting of LG was more efficient in wood species in which there is a relative low amount of S units. G units present higher tendency to addition reaction in comparison with S units, due to the lower content of methoxy groups in *ortho*-position. Otherwise, the radicals produced by laccase in S units should be more stable than those produced in G units, since S units have two electro-donating groups (methoxy) in *ortho*-position instead one methoxy group which possess G units [31]. This extra methoxy group of S units seems to have a remarkable effect in the laccase-mediated polymerization of technical lignins [32]. However, our results suggest that the free *ortho*-position in G units apparently favored the laccase-mediated grafting of LG.

### 3.2. XPS Study

X-ray photoelectron spectroscopy (XPS) analysis was performed to obtain a better knowledge of the surface chemistry of the enzymatically hydrophobized beech veneers. Such technique has been previously used to study the surface chemistry of several wood species [33,34], pulp and paper [35,36], cellulose nanocrystals [37,38] and biocomposites [39,40]. Nevertheless, XPS analysis of some lignocellulosic species, e.g., softwood must be carefully conducted due to the migration of lipophilic extractives to the surface of the material which could be induced by the vacuum conditions necessary to perform the analysis [41]. XPS analysis of beech wood samples provided the elemental composition e.g., carbon (C1s), oxygen (O1s) and nitrogen (N1s), but also the functional groups which are present in the surface of the wood veneers. Thus, the results obtained were a powerful tool to study the changes induced by the laccase-assisted grafting of LG onto beech veneers.

The elemental composition results confirmed that beech samples treated enzymatically with LG showed a clear increase of carbon atoms (4.8%) and a decrease of oxygen (5.9%) which were related to the presence of the aliphatic tail of LG onto the veneers surface (Table 1). In addition, wood samples treated with laccase showed a higher nitrogen content than untreated wood samples which suggests that laccase remained partially adsorbed on the veneers surface even after the washing process. Importantly, adsorption of laccase seems to be reduced when LG is present in the reaction [33].

Four different components were obtained after deconvolution of C1s spectra. C1 was related to C-C or C-H bonds; C2 comprised a carbon bonded to single non-carbonyl oxygen atom (C-O); C3 corresponded to a carbonyl group (C=O) or a carbon bonded to two non-carboxyl oxygen atoms (O-C-O); C4 was assigned to a carbon bonded to a carbonyl group and non-carbonyl oxygen (O-C=O). Beech veneers treated with laccase showed a remarkable increase of C3 component which was related with the oxidation of hydroxyl groups in the phenolic moieties of lignin to form carbonyl groups. The presence of hydrophobic compounds on wood surface could be related with both a low O/C ratio and a high C1/C2 ratio [42]. Samples treated only with laccase showed an important reduction of C-C or C-H bonds and a higher proportion of both ether (C2) and carbonyl (C3) groups which could explain the increase in the hydrophobicity of the laccase-treated wood samples (Figure 2). Samples treated with LG without laccase addition showed a high C1/C2 ratio but also a relative high O/C ratio which means that LG could be adsorbed on the wood surface but not stably bonded. Nevertheless, the LG-treated wood samples did not improve their hydrophobicity (Figure 2) which suggests that the adsorption of LG was not meaningful, or the adsorbed LG was not properly oriented. On the contrary, beech veneers enzymatically treated with LG showed the lowest O/C ratio and a much higher C1/C2 ratio than untreated samples. Therefore, the increase of the C1/C2 ratio (39%) and the decrease of the O/C ratio (25%) suggest that LG was stably bonded onto the wood samples by means of the laccase-mediated treatment.

### 3.3. FT-IR Analysis

**Figure 4:**
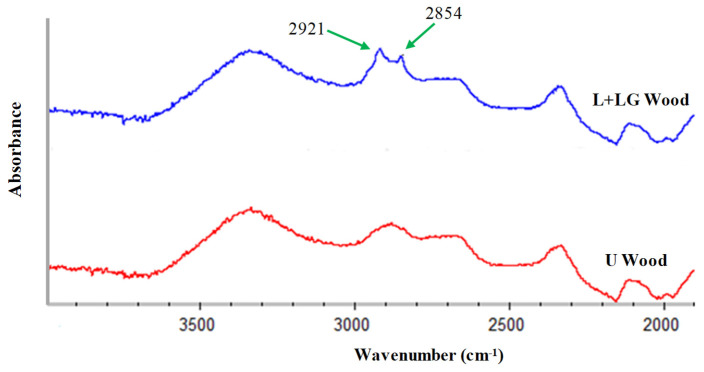
FT-IR spectra between 2000 and 4000 cm^−1^ of untreated beech wood veneers (U Wood) and beech wood treated with laccase and lauryl gallate (L + LG Wood).

Several works have shown that FT-IR spectra could be a useful technique to assess the grafting of hydrophobic compounds onto the surface of biobased materials after their laccase-mediated hydrophobization [18,19,43]. Generally, the main differences between both hydrophobized and unmodified lignocellulosic materials are due to the vibration of the chemical groups which are present in the aliphatic chain of the grafted compound. Thus, FT-IR spectra analysis of the enzymatically hydrophobized beech veneers showed two small peaks at 2921 and 2854 cm^−1^ which were related with the stretching of methyl (-CH_3_) and methylene (-CH_2_-) groups of the 12-carbon aliphatic chain of LG (Figure 4). However, such small peaks were not detected in the spectra of unmodified samples. Therefore, FT-IR spectra analysis evidenced that there was a stable link between LG and beech veneers surface after the laccase-assisted treatment.

### 3.4. Biofilm Assay

The biofilm assay was performed for the assessment of the potential antifouling properties provided by the laccase-mediated grafting of LG onto the surface of pine veneers surface. Pine wood was the specie used for the antifouling assay due to its high hydrophobicity (Figure 3) but also its economic importance in the Atlantic area of Europe. The conditions for the enzymatic grafting of LG were those found in the factorial design (5 mM LG, 5 U of laccase/cm^2^ of wood and 2 h of treatment).

Regarding the antimicrobial properties of LG, [44] found that the antimicrobial activity of alkylphenols was related with the hydrophobicity of the *para*-substituent. In addition, it was proved that LG has antibacterial properties, specifically against Gram-positive bacteria due to inhibition of their membrane respiratory chain [45]. However, the bacterium (*C. marina*) in this study is a Gram-negative bacterium which suggest that the potential antifouling properties of the enzymatically-hydrophobized pine veneers were mostly related with their hydrophobicity. It was showed that the antifouling properties of a substrate are directly linked to its surface energy [46]. It was observed that the lowest surface retention of fouling organisms was achieved when the surface energy of the substrata was between 20–30 mN/m. Since surface energy is inversely proportional to WCA, hydrophobic substrata present low surface energy. In fact, they showed that the atomic groups that performed best antifouling properties were hydrophobic domains such as methyl groups. Thus, it was expected that the long aliphatic chain of LG provided a hydrophobicity high enough to hinder the *C. marina* adhesion onto pine veneers.

At the same time, bacterial surface chemistry is also an important parameter since its hydrophilicity/hydrophobicity character will determine its chemical compatibility and the strength of its initial attachment with the hydrophobized pine veneers. Therefore, the surface chemistry of *C. marina* was studied to assess its hydrophilicity degree. Hence, *C. marina* was suspended in a solution containing both aqueous and organic phases and the migration of the bacteria to the organic phase was measured. According to Karunakaran and Biggs [47], a bacterial migration higher than 50% would mean that bacteria possess a hydrophobic surface. Nevertheless, the partition coefficient was 19.65 ± 2.86% which means that more than 80% of *C. marina* remained in the aqueous phase. Therefore, the surface chemistry of the *C. marina* strain (CECT4278) was mostly hydrophilic.

### 3.5. Protein Measurement

**Figure 5:**
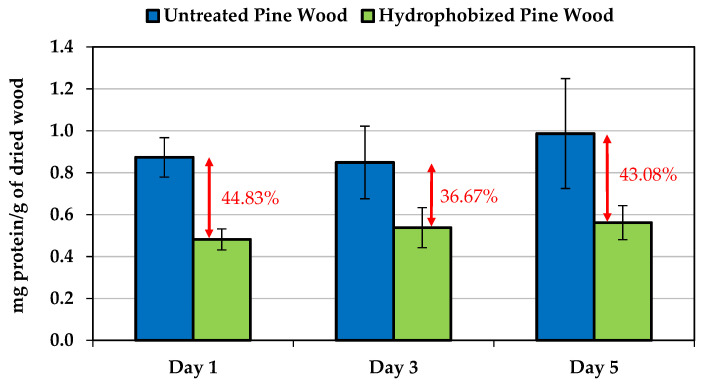
Protein measurement of the hydrophobized pine veneers submerged for 5 days in a marine broth containing a *C. marina* culture.

The potential antifouling properties of the enzymatically-hydrophobized pine veneers were studied by incubating the wood samples in a marine broth inoculated at 3% of *C. marina* (*v*/*v*) for 1, 3 and 5 days. The amount of bacteria onto the veneers surface was indirectly quantified by measuring the protein content on the surface of the wood samples. Such protein measurement of the colonized wood samples showed that the laccase-assisted grafting of LG reduced substantially the *C. marina* adhesion (Figure 5). After one day, the hydrophobized pine veneers showed a protein content of 44.83% lower than untreated veneers. Such trend was also observed after 3 and 5 days of incubation of the wood veneers in the marine broth inoculated with *C. marina*. Apparently, the hydrophobicity induced by the laccase-assisted grafting of LG onto the surface of pine veneers modified the pattern of *C. marina* adhesion, restricting the bacterial attachment and/or the secretion of EPS.

It is worth noting that LG was grafted onto the aromatic moieties of lignin, which means that the hydroxyl groups of cellulose and hemicelluloses were not significantly modified [22]. Therefore, the surface of the enzymatically treated pine veneers resembles to an amphiphilic surface, since both hydrophilic (hydroxyl groups from cellulose and hemicellulose) and hydrophobic (alkyl groups from LG) domains are present on the veneers surface. Such particular chemical composition could hinder the adhesion of a wide range of microorganisms [48,49], but also reduce the impact of the secreted proteins which has an important role in the microorganisms attachment [50,51]. The results proved the efficiency of the laccase-assisted functionalization to provide antifouling properties to wood.

### 3.6. SEM Analyses

The surface of the colonized pine veneers was studied by SEM to confirm the results obtained through the protein measurements. It was expected to detect the EPS produced by *C. marina* after their attachment onto veneers surface [52]. Such EPS are a real problem since they may protect bacteria against biocides and antibiotics [53]. In addition, EPS have chelating properties which are used by bacteria to enhance the nutrients availability [54]. Therefore, by hindering the production of EPS it could be easier to attack the bacteria and reduce their biofouling activity.

**Figure 6:**
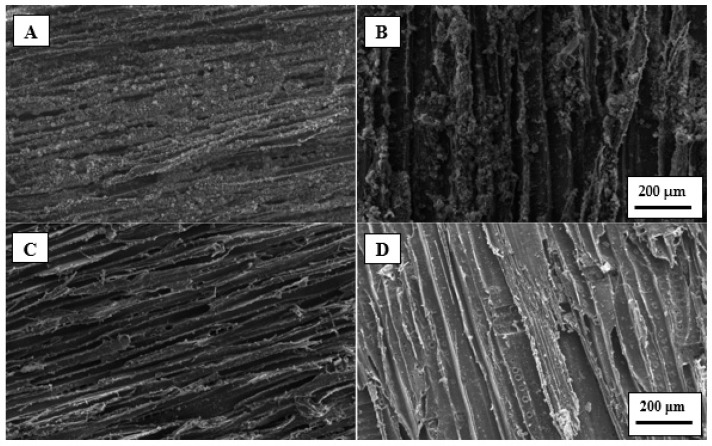
SEM pictures of pine wood veneers colonized by *C. marina*. Untreated pine wood veneers after one day of anti-biofilm assay (**A**); and after five days (**B**). Enzymatically hydrophobized pine wood veneers after one day of anti-biofilm assay (**C**); and after five days (**D**). All the pictures were acquired at 100× magnification.

Untreated pine veneers showed a relatively high density of EPS after one day of in-cubation in the marine broth and, such EPS density was even higher after the fifth day of test (Figure 6A,B). These pictures suggest that C. marina can adhere easily to the surface of untreated pine veneers. On the contrary, SEM pictures of the enzymatically hydrophobized pine veneers showed a much lower density of EPS compared with untreated veneers. Moreover, there were not significant differences in the apparent EPS density of the hydrophobized pine veneers between the first and the fifth day of incubation in the marine broth. These results agreed with the data obtained in the measurements of the amount of protein which was present on the veneers surface. There are two plausible mechanisms that could explain the big differences observed between the SEM pictures of the untreated and the enzymatically hydrophobized pine veneers. One the one hand, the 12-carbons aliphatic tails of the LG that was enzymatically grafted onto the surface of pine veneers could disrupt notably the normal secretion of EPS. On the other hand, the hydrophobization of the pine veneers could delay or directly hinder the surface colonization of *C. marina* and, therefore the EPS could not be secreted yet.

**Figure 7:**
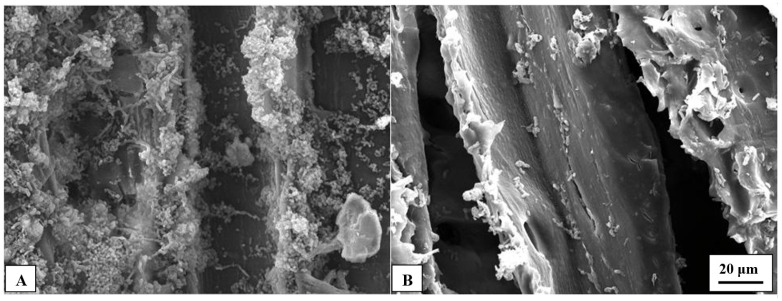
SEM pictures of colonized pine wood veneers after five days of antibiofilm assay. Untreated pine wood veneers (**A**); and enzymatically hydrophobized pine wood veneers (**B**). The pictures were acquired at 1000× magnification.

It is worth noting that both untreated and hydrophobized pine veneers showed an important surface roughness which apparently affected to the bacterial adhesion. In fact, the higher density of EPS was observed on the angled edges of the veneers surface, which means that such angled zones likely favored the attachment of *C. marina* (Figure 7). Nonetheless, hydrophobized pine veneers showed a much lower density of EPS onto such angled edges than untreated veneers which, confirm the effectiveness of the enzymatic grafting of LG. These results confirm that the laccase-assisted grafting of LG modified the normal pattern of *C. marina* adhesion and/or the secretion of EPS. Thus, the marine antifouling properties of the pine veneers were remarkably improved by means of the laccase-assisted grafting of LG onto the pine veneers surface. These results could lead to the development of a new environmentally friendly treatment to protect wood in marine environments.

## 4. Conclusions

A new environmentally friendly strategy to improve the marine antifouling properties of wood has been proposed in the present study. Such new green strategy is based on the laccase-assisted grafting of LG onto wood veneers. Different wood species (beech, pine, and eucalyptus) were effectively hydrophobized through the enzymatic treatment. It was observed that the reaction conditions played an important role on the extent of hydrophobization, but the treated wood species were also a major factor. Based on these results, pine wood was selected to study the impact of the laccase-mediated hydrophobization on the marine antifouling properties of wood. SEM pictures and protein measurements confirmed that the hydrophobized wood veneers modified the colonization pattern of *Cobetia marina*, revealing that the proposed enzymatic methodology could act as a new marine antifouling treatment for lignocellulosic materials. Future studies should analyze the antifouling properties of the enzymatically treated wood against other marine microorganisms and the “in situ” response of the hydrophobized wood under marine environments. The combination of the proposed treatment with other conventional ones is also of interest for future research.

## Figures and Tables

**Table 1:** Elemental quantification (%) and functional groups relative percent (%) of beech veneers. Untreated beech wood veneers (UW); beech veneers treated with lauryl gallate (LG); treated with laccase (L); treated with laccase and lauryl gallate (L + LG).

Sample	Elements			C1s Components				O1s Components		O/C Ratio	C1/C2 Ratio
	C1s	O1s	N1s	C1%	C2%	C3%	C4%	O1%	O2%		
UW	65.29	30.85	1.29	47.25	38.98	11.30	2.47	16.82	83.19	0.48	1.21
LG	66.60	29.06	1.72	53.79	30.63	11.05	4.54	17.30	82.71	0.44	1.76
L	65.39	26.75	6.42	38.70	42.46	16.60	2.25	30.40	69.61	0.41	0.91
L + LG	70.09	24.95	3.24	53.68	31.99	11.77	2.56	26.68	73.32	0.36	1.68

## Data Availability

The data presented in this study are available on request from the corresponding author.

## Supplementary Materials

The following are available online at https://www.mdpi.com/article/10.3390/polym13213795/s1, Table S1. Treatment conditions for the enzymatic grafting of lauryl gallate (LG) on beech veneers tested in the experimental design.
