# Supplementary material for: Enzymatic Functionalization of Wood as an Antifouling Strategy against the Marine Bacterium Cobetia marina
Source: Polymers (Basel). 2021 Nov 2;13(21):3795. doi: 10.3390/polym13213795 (PMC8587834; doi:10.3390/polym13213795)
Supplement: Supplementary file 1 [file polymers-13-03795-s001.zip › polymers-1430262-supplementary.pdf]

Enzymatic functionalization of wood as an antifouling strategy against the marine bacterium *Cobetia marina*

Daniel Filgueira [1, 2], Cristian Bolaño [1], Susana Gouveia [1], Diego Moldes [1, *]

[1] CINTECX, Department of Chemical Engineering, Campus Universitario As Lagoas-Marcosende, Universidade de Vigo, E-36310, Vigo, Spain; damfilgueira@hotmail.com; cbolaño@uvigo.es; gouveia@uvigo.es

[2] TECNALIA, Basque Research and Technology Alliance (BRTA), Area Anardi 5, 20730, Azpeitia, Spain; daniel.martinez@tecnalia.com

* Research Group of Bioengineering and Sustainable Processes, Department of Chemical Engineering, Edificio Fundición, Lagoas Marcosende s/n, University of Vigo, 36310, Vigo (Spain); diego@uvigo.es

Corresponding author: diego@uvigo.es

Contents

2 pages

1 Table

TABLES

Table S1. Treatment conditions for the enzymatic grafting of lauryl gallate (LG) on beech veneers tested in the experiment design.

[LG] mM	Laccase dose (U/cm^2 wood)	Time of reaction (h)
5	6.72	2
5	5.0	2
2.5	5.0	4
5	3.36	1
5	6.72	4
5	3.36	2
5	3.36	4
2.5	6.72	1
2.5	6.72	2
10	6.72	4
5	5.0	4
2.5	3.36	2
5	6.72	1
10	3.36	2
10	3.36	4
2.5	6.72	4
2.5	3.36	1
10	3.36	1
10	5.0	1
10	5.0	4
2.5	5.0	1
2.5	3.36	4
10	5.0	2
10	6.72	1
5	5.0	1
2.5	5.0	2
10	6.72	2
